# Malignant features related PRDX1 associated with osimertinib sensitivity of EGFR-mutant lung adenocarcinoma

**DOI:** 10.7150/ijms.107255

**Published:** 2025-03-31

**Authors:** Wenying Jiang, Maonan Wang, Xiaoqian Yu, Guoqian Liu, Xiaoyun He, Cheng Mei, Chunlin Ou

**Affiliations:** 1 Department of Pathology, Xiangya Hospital, Central South University, Changsha 410008, Hunan, China.; 2 Department of Pathology, Xiangya Hospital, School of Basic Medical Sciences, Central South University, Changsha 410000, Hunan, China.; 3 Departments of Ultrasound Imaging, Xiangya Hospital, Central South University, Changsha 410008, Hunan, China.; 4 Department of Blood Transfusion, Xiangya Hospital, Clinical Transfusion Research Center, Central South University, Changsha 410008, Hunan, China.; 5 National Clinical Research Center for Geriatric Disorders, Xiangya Hospital, Central South University, Changsha, China.

**Keywords:** PRDX family, lung adenocarcinoma, prognosis, immune infiltration, immunotherapy, therapeutic target

## Abstract

The peroxiredoxin (PRDX) family, also known as the peroxidase family, consists of six members that participate in a variety of essential bio-processes in carcinogenesis. However, their molecular role in lung adenocarcinoma (LUAD) has not been systematically explored. Using bioinformatic tools, we systematically analyzed the expression, prognostic value and drug sensitivity of the PRDX gene family members in LUAD. Quantitative real-time polymerase chain reaction (qRT-PCR) was performed to verify the expression of PRDX1 in both LUAD tissues and cells. Cell Counting Kit-8 (CCK-8) assay was applied to detect the half-maximal inhibitory concentration (IC_50_) of osimertinib in LUAD. A series of cellular drug assays, including 5-Ethynyl-2'-deoxyuridine (EdU), colony formation, and apoptosis assays, were performed to explore the correlation of PRDX1 with epidermal growth factor receptor-tyrosine kinase inhibitor (EGFR-TKI) sensitivity by using EGFR-mutant and wild-type LUAD cell lines. Among all the PRDX family members, PRDX1 has a promising prognostic value and is associated with EGFR mutations, as verified by experiments conducted on collected LUAD specimens. In addition, pathway enrichment analysis suggested that PRDX1 expression positively correlated with DNA repair, which is often considered to be inextricably linked to drug resistance in tumor cells. Thus, we validated the correlation between PRDX1 and EGFR-TKI sensitivity through a series of *in vitro* experiments and found that PRDX1 inhibition along with osimertinib treatment resulted in synergistic inhibition of tumor growth. Moreover, we found that PRDX1 was negatively correlated with the immune infiltration of dendritic cells (DCs) in the tumor microenvironment (TME) of LUAD, further suggesting an oncogenic role of PRDX1. This study demonstrates that high PRDX1 expression could be a potential diagnostic and prognostic marker of LUAD, and the strategy of PRDX1 knockdown provides new insights into improving the therapeutic sensitivity of EGFR-TKI in patients with LUAD.

## Introduction

Lung cancer is one of the most lethal malignancies that significantly endangers human health 1. Non-small cell lung carcinoma (NSCLC) is the most prevalent histological type of lung cancer, comprising approximately 85% of all lung cancer cases 2. NSCLC can be further classified into three subtypes: lung adenocarcinoma (LUAD), squamous cell carcinoma (SCC), and large-cell carcinoma 1. LUAD is the most common subtype within NSCLC, accounting for approximately 40% of the global lung cancer incidence 3. It is characterized by early-stage micrometastasis and a dismal 5-year survival rate ranging from 5-20% 4. Additionally, owing to tumor heterogeneity, drug resistance and recurrence are frequently encountered during the treatment of patients with LUAD 5, 6. Therefore, there is an urgent need to identify additional tumor markers and therapeutic targets to assist in the diagnosis, treatment, and prognosis improvement of LUAD.

The peroxiredoxin (PRDX) family comprises six members, including PRDX1, PRDX2, PRDX3, PRDX4, PRDX5, and PRDX6 7. Previous studies 8, 9 have shown that members of the PRDX family may serve as biomarkers with good prognostic value in various tumors. However, the role of this gene family in LUAD progression remains unclear. In this study, we comprehensively explored the prognostic and diagnostic significance of PRDX family members in LUAD by examining their expression patterns, clinical characteristics, prognosis implications, and drug sensitivity, subsequently focusing on PRDX1. Aberrant expression of PRDX1 has been identified in a variety of tumors and pre-cancerous lesions, including oral squamous cell carcinoma (OSCC) and cervical carcinoma 10, 11. Its high expression safeguards tumor cells from oxidative stress-induced damage and enhances tumor resistance to radiotherapy, which is closely associated with tumor recurrence and poor prognosis 12, 13.

Our study demonstrated a correlation between PRDX1 and epidermal growth factor receptor (EGFR) mutations and tyrosine kinase inhibitor (TKI) sensitivity. These associations were preliminarily verified through *in vitro* experiments. Overall, our findings suggest that PRDX1 could serve as a potential prognostic marker for LUAD. Moreover, targeting PRDX1 may offer novel perspectives on enhancing the efficacy of EGFR-TKI therapy in patients with LUAD.

## Materials and methods

### Expression analysis of the PRDX family

TIMER2.0 (http://timer.cistrome.org/) is a comprehensive web server based on The Cancer Genome Atlas (TCGA). It allows for the comparison of target gene expression differences between tumor tissues and adjacent normal tissues 14. Utilizing TIMER2.0, which contains samples from 515 LUAD and 59 normal lung tissues, we investigated the mRNA expression of PRDX family members across all TCGA cancer types or specific cancer subtypes. The expression values are normalized as log_2_-transformed transcripts per million (TPM).

Additionally, we made use of UALCAN (http://ualcan.path.uab.edu), a publicly accessible platform for exploring mRNA expression, clinicopathological features, and DNA methylation in various malignancies. We employed this platform to compare the mRNA expression of the PRDX family between 515 LUAD samples and adjacent normal 59 lung tissues 15.

Furthermore, protein levels of PRDX family members in LUAD and pulmonary tissues were validated using immunohistochemistry (IHC) data from the Human Protein Atlas (HPA) database (http://www.proteinatlas.org/). The HPA contains a variety of IHC images sourced from 20 types of common human cancers 16

### Analysis of clinicopathological features

We utilized UALCAN to analyze the mRNA expression profiles of PRDX family members based on individual cancer stages and nodal metastasis status in LUAD. In addition, by obtaining RNA sequencing (RNA-seq) data of 598 LUAD samples from TCGA database, we constructed logistic regression models for each PRDX gene using the R 'stats' package to evaluate their discriminatory effects on clinical variables (T stage, N stage, M stage, sex, age, and smoking status). The TCGA-LUAD transcript data were normalized using the log_2_ (TPM+1) transformation method.

### Survival analysis

We employed the Kaplan-Meier Plotter (https://kmplot.com), a publicly available website tool for survival analysis, to analyze the correlation between the mRNA expression of PRDX family members and LUAD patient prognosis 17. The 1,308 LUAD samples were stratified into high- and low-expression groups for each PRDX gene using median mRNA expression levels as the cutoff. Prognostic significance was assessed by log-rank tests, with a *P*-value < 0.05 considered statistically significant. In addition, based on the TCGA-LUAD transcript data, we performed univariate Cox regression analyses to validate the prognostic value of each PRDX family member, and the R package 'survival' was used for proportion risk hypothesis testing. Transcript data normalization followed the log_2_(TPM + 1) method.

### Comparison of drug sensitivity, exploration of the expression of PRDX genes in EGFR-mutant LUAD, and molecular docking

We leveraged the expression profiles of the PRDX family to analyze drug sensitivity data for the top 30 compounds from the Cancer Therapeutics Response Portal (CTRP). These data were retrieved via the Gene Set Cancer Analysis (GSCA) platform to compare pan-cancer drug responses. The GSE31210 dataset encompasses 127 patients with EGFR mutations, 20 with KRAS mutations, 11 with EML4-ALK fusions, and 68 triple-negative LUAD cases 18. Gene expression was normalized using the z-score model. The Wilcoxon rank-sum test was used to analyze the differences in the expression of PRDX1, PRDX2, and PRDX3 between EGFR-mutant LUAD samples and EGFR wild-type samples. A *P*-value < 0.05 was regarded as statistically significant.

In addition, we obtained the 3D structure of PRDX1, determined by X-ray diffraction, from the RCSB PDB database (https://www.rcsb.org/) 19 Subsequently, we processed this protein file using PyMOL. The 3D-structure of osimertinib in SDF format was obtained from PubChem (https://pubchem.ncbi.nlm.nih.gov) 20 and converted into the mol2 format using the OpenBabel software v2.4.1 21. Then, we performed molecular docking of the PRDX1 protein complexed with the ligand osimertinib using Autodock4 22. The prediction results were visualized using the PLIP web tool (https://plip-tool.biotec.tu-dresden.de) 23, PyMol (DeLano WL [2002] PyMOL molecular graphics system. http://www.pymol.org), and the DiscoveryStudio_v4.5 software.

### Methylation and mutation analysis

To investigate the association between PRDX1 expression levels and DNA methylation, we used the GSCA website tool (http://bioinfo.life.hust.edu.cn/) 24. Additionally, the mutation analysis of PRDX1 was conducted using cBioportal (http://www.cbioportal.org/).

### Enrichment pathway analysis

cBioportal is a comprehensive and publicly accessible platform using which we retrieved a set of genes co-expressed with PRDX1 from the TCGA database 25. The 'ggplot2' package in R was utilized to generate a volcano plot for the genes co-expressed with PRDX1. By setting a |log_2_FC| threshold of 1.8 and a *P*-value cutoff of 0.05, we identified the 84 key genes co-expressed with PRDX1. Subsequently, their interactions were analyzed using the web-server STRING (https://cn.string-db.org/). A detailed visualization was performed using Cytoscape v3.9.1 26. In addition, genes significantly correlated with PRDX1 expression (*P* < 0.05) underwent ID conversion using the R package 'org.Hs.eg.db'. These genes were further analyzed through Gene Ontology (GO) and Kyoto Encyclopedia of Genes and Genomes (KEGG) enrichment analysis using the R package 'clusterProfiler'. The results of Gene Set Enrichment Analysis (GSEA) obtained from the R package 'clusterProfiler' were also used to quantitatively evaluate the functional enrichment of PRDX1. Specifically, when the normalized enrichment score (NES) was positive, genes were positively associated with the pathway; conversely, when the NES was negative, genes were negatively associated with the pathway.

### Immune infiltrating analysis

TISCH (http://tisch.comp-genomics.org/) is an online database that comprehensively collects tumor single-cell transcriptomic profiling data, with a particular focus on the tumor microenvironment (TME) 27. We obtained information on the infiltration of PRDX1 into LUAD TME cells from the GSE131907 dataset. This dataset consisted of single-cell RNA-seq data for 208,506 cells derived from 58 LUAD samples. In addition, by utilizing TIMER2.0, we evaluated the correlation between the mRNA expression of PRDX1 and immunoinfiltrating cells in LUAD, including B cells, M2 macrophages, monocytes, plasma cells, and dendritic cells (DCs). Furthermore, GEPIA2.0 (http://gepia2.cancer-pku.cn/) was utilized to assess the correlation between PRDX1 expression and DC-specific immune markers 28. Spearman's correlation coefficients were adjusted for purity. *P*-value < 0.05 was considered statistically significant.

### Sample collection, RNA extraction, and detection

A total of 25 pairs of matched paracancerous normal tissue samples and paraffin-embedded archival LUAD specimens were obtained from Xiangya Hospital (Changsha, China). These 25 pairs of LUAD samples included 12 pairs of EGFR wild-type and 13 pairs of EGFR-mutant type (exon 21 mutations) samples. None of the patients had received any form of therapy, such as chemotherapy, radiotherapy, or immunotherapy, before resection. The Research Ethics Committee of Xiangya Hospital of Central South University approved the collection of clinical LUAD specimens.

Total RNA from formalin-fixed paraffin-embedded (FFPE) LUAD or normal samples was isolated and extracted using total RNA AmoyDx® FFPE RNA Extraction Kit (Cat. #8.02.0019; AmoyDx, Xiamen, P. R. China). RNA was extracted from LUAD cell lines and normal lung epithelial cells using an RNA Simple Total RNA Kit (TIANGEN, China). RNA amplification and qRT-PCR were performed as previously described 29, 30. The quantitative real-time polymerase chain reaction (qRT-PCR) primer sequences are presented in **Table 1**.

### Cell culture

Normal human lung epithelial cells (BEAS-2B) and LUAD cell lines (A549, PC-9, and H1299) used in the experiments were obtained from the Cell Bank of the Type Culture Collection of the Chinese Academy of Sciences. All cell lines were cultured in RPMI 1640 medium supplemented with 10% fetal bovine serum (FBS) and 1% penicillin/streptomycin in a 5% CO_2_ humidified incubator at 37℃. All cells were free of *Mycoplasma*, other bacteria, and fungi.

### Small interfering RNA (siRNA) assay and cell transfection

The siRNA for PRDX1 was commercially obtained from Ribobio (China). Lipofectamine 3000 was used as the transfection reagent, and the interference sequence was siPRDX1: 5′-ATGAACATTCCTTTGGTAT-3′. LUAD cells were seeded into 6-well plates. Transfection was carried out once the cell density reached a minimum of 70%. The detailed procedures of cell transfection have been previously described by Feng et al. 31.

### Detection and analysis of half-maximal inhibitory concentration (IC_50_) of osimertinib in LUAD cells

Cells were seeded at a density of 5 × 10^4^ cells per well in a 96-well plate. They were then incubated in a 5% CO_2_ incubator at 37℃ for 24 h. Multiple wells were set up in parallel to ensure accuracy. Based on the cell sensitivity to osimertinib, we established different concentration gradients by incubating the cells for 48 h to determine the IC₅₀ of osimertinib in A549 and PC-9 cell lines. Subsequently, 200 μL of 10% Cell Counting Kit-8 (CCK-8) reagent was added to each well for detection, and the plate was incubated for an additional 2 h. The absorbance was measured at an optical density (OD) value of 450 nm using a microplate reader. After excluding data with significant differences within each group, the average values were used to calculate the growth inhibition rate. The formula for calculation was: Growth Inhibition Rate = 1 - (A_osimertinib group - A_solution zeroing) / (A_blank group - A_blank zeroing), where "A" represents absorbance. The experiment was repeated three times. The IC₅₀ values were analyzed and calculated using SPSS version 23.0.

### 5-Ethynyl-2'-deoxyuridine (EdU) assay

A total of 5 ×10^4^ transfected cells were seeded into 96-well plates and incubated at 37℃ for 24 h. The experiment was conducted using the RiboBio EdU kit (C10310-3) in strict compliance with the manufacturer's instructions.

### Colony formation assay

For the colony formation assay, 1 × 10^3^ cells from each experimental group were seeded into 6-well plates and incubated at 37℃ for 10 days. Subsequently, the cells were washed with phosphate-buffered saline (PBS) and fixed with methanol for 15 min. After fixation, the cells were stained with a 1% crystal violet solution for 30 min at room temperature.

### Apoptosis assay

Forty-eight hours post-transfection, cells from different treatment groups were harvested for apoptosis assay, which was performed using the AP101-100 kit (MULTI SCIENCE). Samples were prepared in accordance with the kit's instructions, and flow cytometry was used to identify and quantify apoptotic cells.

### Statistical analysis

Statistical analyses were carried out using GraphPad Prism 7 and SPSS 23.0. All experiments were repeated in triplicate. The Student's *t*-test was used to compare two sets of data. For all analyses and visualizations of TCGA data, including regression and co-expression gene enrichment analyses, R 4.2.1 was utilized. Survival analysis was performed using the log-rank test. Spearman's correlation was applied to evaluate the association among the PRDX family, immune regulators, and immunological infiltration. The prognostic model was visualized using forest plots by the R packages 'ggplot2' and 'survival'. All statistical tests were two-sided, and* P* < 0.05 was considered statistically significant.

## Results

### Expression analysis of PRDX family members in LUAD

**Figure 1:**
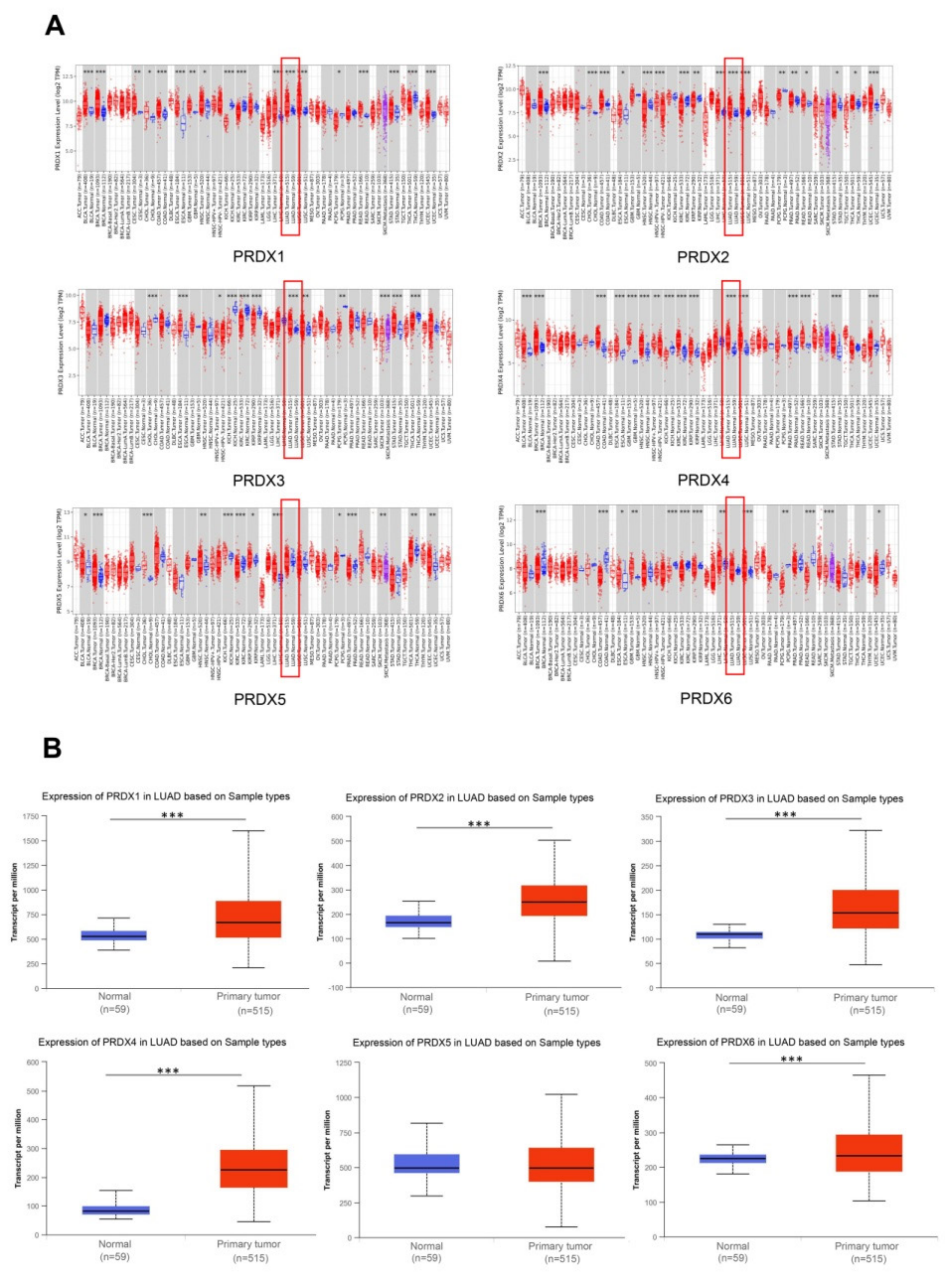
Expression of PRDX family members in LUAD. (A) Expression of each PRDX family gene in TCGA tumors and adjacent normal tissues analyzed by TIMER2.0. (B) Expression of the PRDX genes in TCGA-LUAD obtained from UALCAN website. *P < 0.05, **P < 0.01, ***P < 0.001 compared with control.

To compare the expression of the PRDX family members in various malignant tumors and adjacent normal tissues, we utilized TIMER2.0 to explore their transcriptional levels. Compared to normal lung tissues, the mRNA expression of PRDX1, PRDX2, PRDX3, and PRDX4 was significantly upregulated in LUAD. However, there was no significant difference in the expression of PRDX5 and PRDX6 (**Figure 1A**).

**Figure 2:**
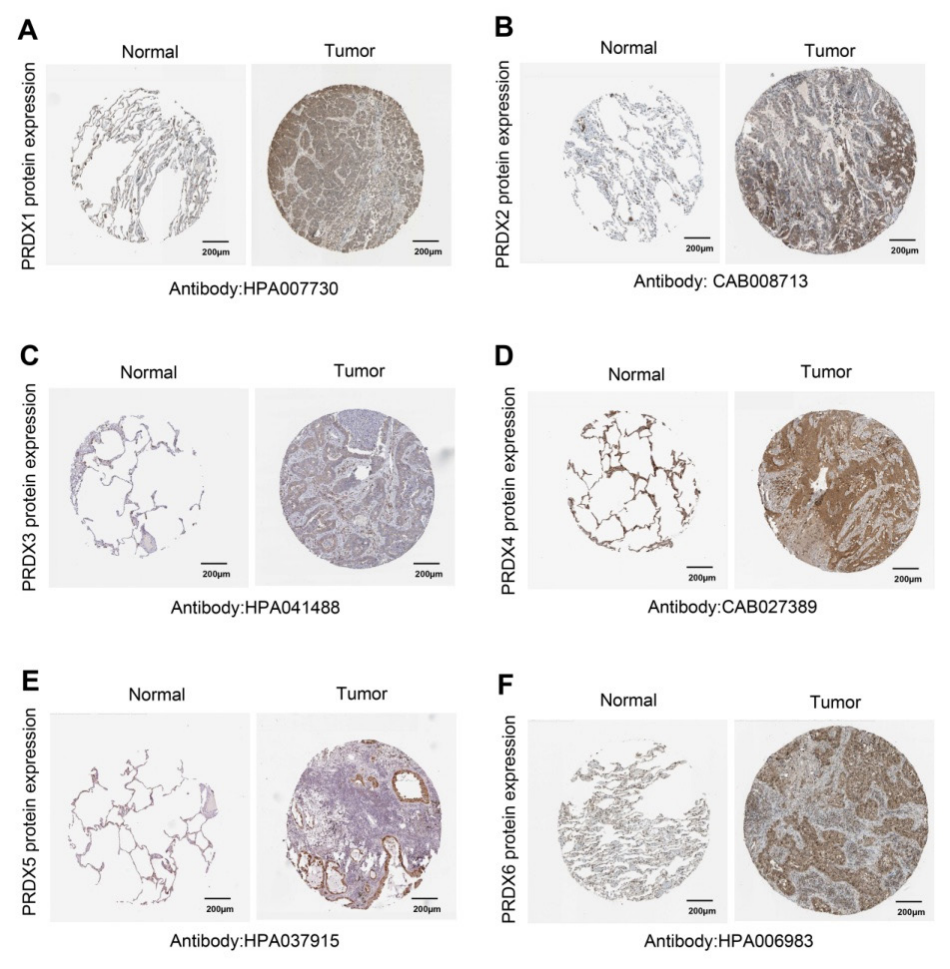
Expression of PRDX genes in LUAD at protein level. (A-F) IHC images were acquired using HPA, which concerns protein levels of PRDX1, PRDX2, PRDX3, PRDX4, PRDX5, and PRDX6 expressed in LUAD and normal samples.

The UALCAN database was then utilized to investigate the differences in mRNA expression of PRDX family members in TCGA-LUAD. The results showed that, except for PRDX5, all PRDX family genes were significantly overexpressed in LUAD compared to normal lung tissue (**Figure 1B**). To further validate the protein levels of PRDX genes in LUAD, we obtained IHC staining profiles from the HPA database. **Figure 2** demonstrated that PRDX1, PRDX2, PRDX3, PRDX5, and PRDX6 were highly expressed in LUAD samples compared to normal samples. Nevertheless, no significant difference was observed in the expression of PRDX4 between LUAD and normal tissues. These findings imply that the high expression of PRDX1, PRDX2, PRDX3, and PRDX6 is closely associated with the progression of LUAD.

### Relationship between the mRNA expression of PRDX family members and clinicopathological features in LUAD

**Figure 3:**
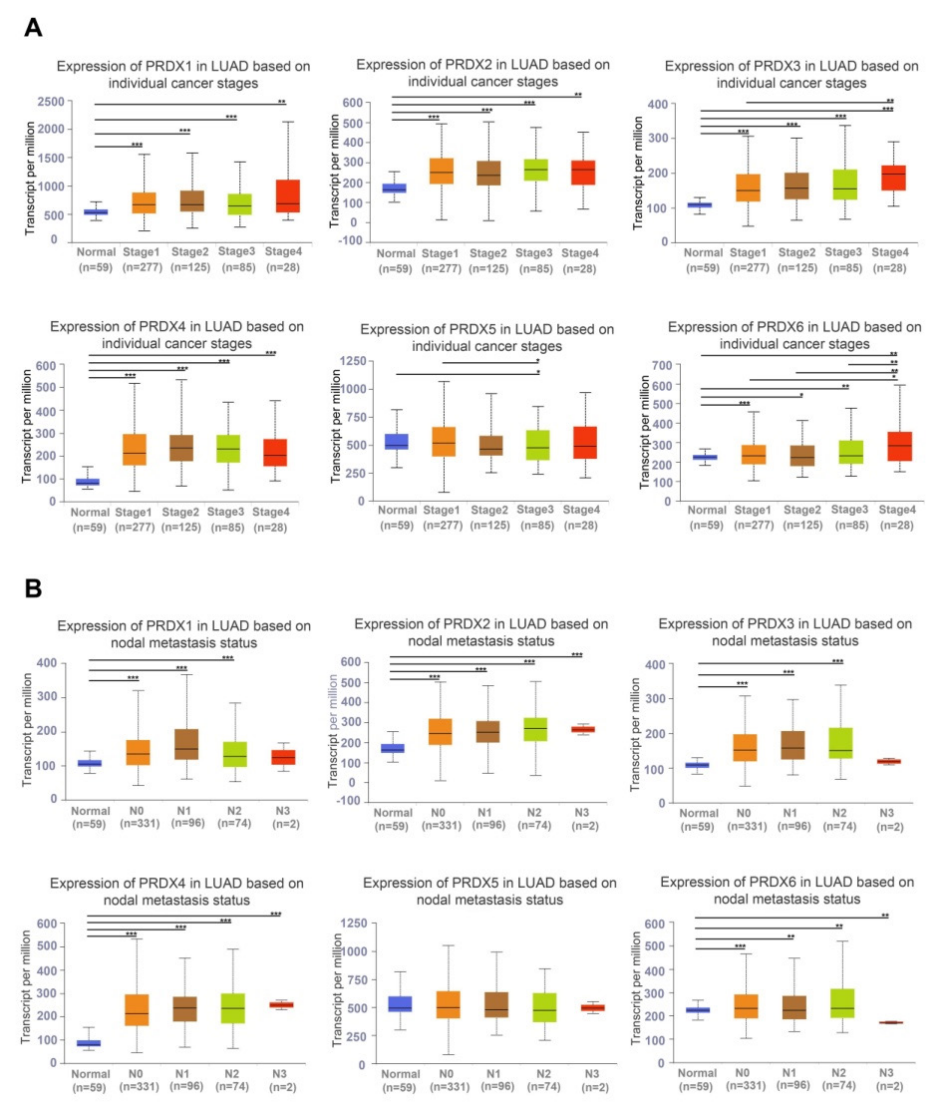
Relationship between the expression of PRDX family genes and clinicopathological features in LUAD based on TCGA data. (A) Expression of PRDX family genes in normal samples and LUAD tissues at different stages. (B) Expression of PRDX genes in normal samples and LUAD samples with N0-N3 lymph node metastasis status. *P < 0.05, **P < 0.01, ***P < 0.001 compared with the control.

Based on a survey of the UALCAN database, PRDX1, PRDX2, PRDX3, PRDX4, and PRDX6 were found to be highly expressed in all LUAD stages. The mRNA expression of PRDX5 was lower in LUAD stage 3 than in stage 1, and there was no difference from normal lung tissue in other stages of LUAD (**Figure 3A**). These results indicate that the expression of PRDX family members significantly correlated with individual cancer stages in LUAD. In all stages of lymph node metastasis, the mRNA expression of PRDX2, PRDX4, and PRDX6 was higher in LUAD samples than in normal samples. The mRNA expression of PRDX1 and PRDX3 was not significantly different between normal lung and stage N3 LUAD tissues. The expression of PRDX5 in normal lung tissues was not significantly different from that in LUAD stage N0-3 (**Figure 3B**). Logistic regression analysis was conducted to explore the correlation between each member of the PRDX family and clinical characteristics of LUAD. The results showed that high PRDX1 expression was associated with T and N stages (**Supplementary Figure 1A**), high PRDX2 expression was correlated with N stage and smoking (**Supplementary Figure 1B**), high PRDX3 and PRDX4 expression was associated with T stage and sex (**Supplementary Figure 1C, D**), upregulated PRDX5 expression was related to smoking (**Supplementary Figure 1E**), and upregulated PRDX6 expression was associated with distant metastasis and sex (**Supplementary Figure 1F**). These findings suggest that the expression of the PRDX family members, excluding PRDX5, could be a potential diagnostic indicator for patients with LUAD.

### Prognostic value of PRDX family members in LUAD

**Figure 4:**
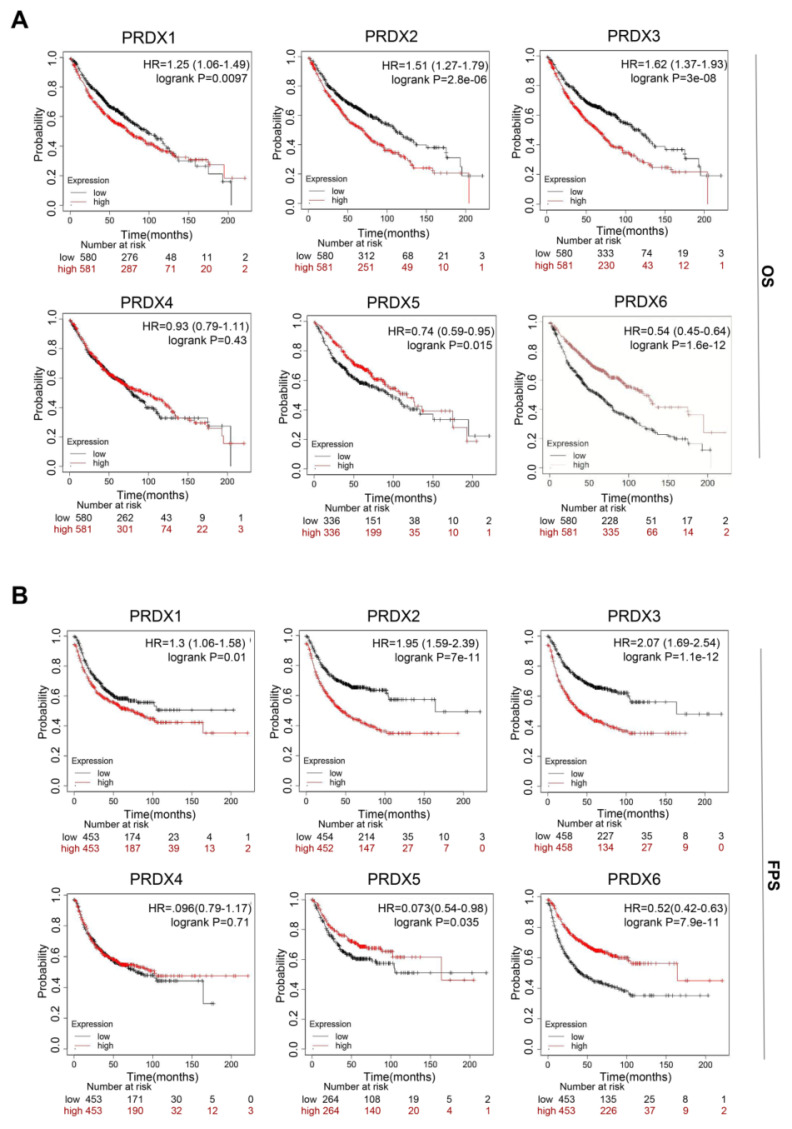
Prognostic analysis of PRDX family genes in patients with LUAD. (A-B) Statistical association of mRNA expression of PRDX genes with OS and FPS in LUAD determined using log-rank tests.

We utilized the Kaplan-Meier database to investigate the prognostic value of each PRDX gene. Specifically, we explored the association between their expression and two survival endpoints: overall survival (OS) and first progression survival (FPS). The results indicated that high expression of PRDX1, PRDX2, and PRDX3 in patients with LUAD was significantly associated with poor OS and FPS. However, overexpression of PRDX5 and PRDX6 was associated with better OS and FPS (**Figure 4A**, **B**). In addition, univariate regression analysis of TCGA-LUAD data showed that TNM stage, PRDX1, PRDX3, and PRDX6 were independent prognostic risk factors for OS in patients with LUAD (**Supplementary Figure 2**). Notably, Kaplan-Meier analysis indicated that high PRDX6 expression was associated with better OS, whereas Cox regression analysis suggested the opposite trend. This inconsistency might be attributed to limited sample size or confounding variables. In summary, the expression of the PRDX family members is closely associated with the prognosis of patients with LUAD. Among these, PRDX1, PRDX2, and PRDX3 are potential prognostic markers of LUAD.

### Comparison of drug sensitivity among members of PRDX family, molecular docking, and exploration of PRDX1 expression in EGFR-mutant LUAD

**Figure 5:**
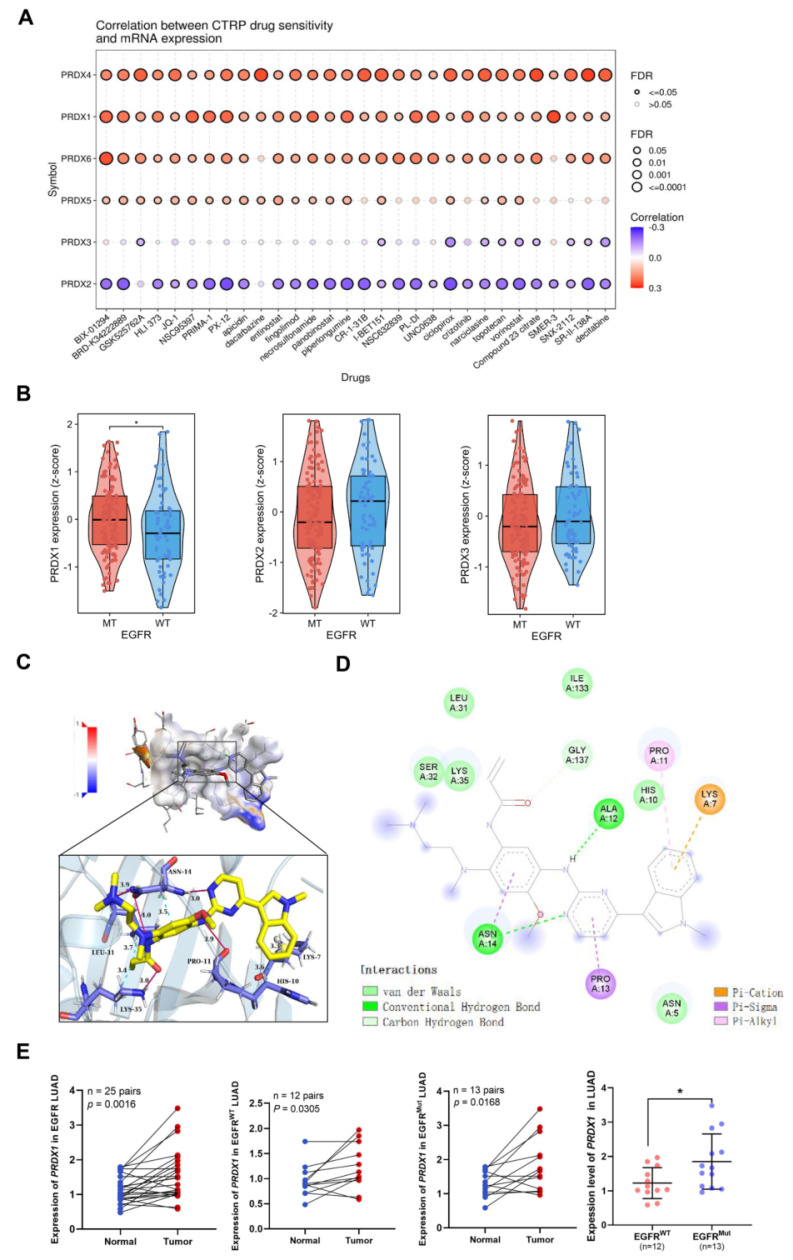
Drug sensitivity comparison, molecular docking, and expression exploration of PRDX1. (A) CTRP drug sensitivity profiles are illustrated from the top to the bottom according to the correlation between each PRDX member and the IC_50_ of each CTRP drug. (B) Expression of PRDX1-3 in EGFR wild-type and mutant LUAD based on the GSE31210 dataset. (C) Visualization of the pocket structure and spatial arrangement of PRDX1 interaction with osimertinib. (D) Energy map of the interaction between PRDX1 and osimertinib. (E) Expression levels of PRDX1 in 25 EGFR LUAD samples, 13 EGFR-mutant samples, and 12 EGFR wild-type LUAD samples compared with that in the adjacent lung tissue, and expression of PRDX1 in the EGFR-mutant samples compared with the EGFR wild-type samples. *P < 0.05 compared with control.

We conducted a comparison of drug sensitivity among the PRDX family members by accessing the GSCA website. As shown in **Figure 5A**, the expression of PRDX1, PRDX4, and PRDX6 was positively correlated with the IC_50_ of CTRP drugs (top 30), suggesting their role in chemoresistance. In contrast, the mRNA expression of PRDX2 was negatively correlated with the IC_50_ of most CTRP drugs. suggesting that its high expression may be associated with drug sensitization. However, the expression of PRDX5 and PRDX3 exhibited only a weak correlation with the IC_50_ of CTRP drugs, suggesting that their influence on drug sensitivity was relatively limited. In conclusion, most PRDX family members showed high resistance to CTRP drugs, especially BIX-01294. BIX-01294 is an inhibitor of G9a histone methyltransferase, which promotes apoptosis in EGFR-mutant LUAD cells 32. This triggered our interest in exploring the association between the expression of PRDX family members and EGFR mutations in LUAD. Subsequently, we acquired the GSE31210 dataset and examined the correlation between EGFR mutations and PRDX family members with good prognostic value (including PRDX1, PRDX2, and PRDX3). The results revealed that PRDX1 was highly expressed in EGFR-mutant LUAD compared to wild-type LUAD, whereas the expression of the other two members did not differ statistically (**Figure 5B**). Integrating the results of expression and prognostic analyses of the PRDX family, we identified PRDX1 as a potential diagnostic and prognostic marker for LUAD. Further exploration of its role in the progression of LUAD was warranted.

Osimertinib, a representative EGFR-TKI, is extensively utilized in the treatment of EGFR-mutant LUAD 33. We employed Autodock4 to compute the docking score between PRDX1 and osimertinib, which was determined to be -5.83. This indicated a robust binding ability between the two entities. **Figure 5C** presents the prediction and visualization of the interaction pocket structure involved in the docking of PRDX1 with osimertinib. These analyses were carried out using PyMol and DiscoveryStudio_v4.5, in collaboration with the PLIP website, which showed the corresponding predicted and visualized spatial arrangement. **Figure 5D** depicts the resultant plot of the simulated interaction of the two molecules. It clearly illustrates that hydrogen bonding, hydrophobic forces, and π-bonding promote and participate in the interaction between PRDX1 and osimertinib, suggesting that they can form a stable complex. Given the important role of EGFR mutations in LUAD development, we collected tissue samples from patients with EGFR-mutant and wild-type LUAD and performed qRT-PCR to detect the expression of PRDX1. The results demonstrated that PRDX1 expression was higher in 25 LUAD samples than in the adjacent normal tissues. Moreover, mRNA expression of PRDX1 was significantly elevated in EGFR-mutant LUAD samples (n=13) when compared to wild-type cases (n=12; *P* < 0.05) (**Figure 5E**). These results imply that PRDX1 is associated with EGFR mutations and may potentially serve as a target for enhancing the sensitivity of patients with EGFR mutations to EGFR-TKI therapy.

### Co-expressed genes and pathway enrichment of PRDX1 in LUAD

Gene mutations play a crucial role in promoting carcinogenesis. We utilized the GSCA database to explore the association between DNA methylation and PRDX1 mRNA expression in patients with LUAD. The corresponding values were evaluated using the Spearman algorithm. Moreover, PRDX1 methylation levels were negatively correlated with mRNA expression, indicating the oncogenic potential of PRDX1 in LUAD (**Supplementary Figure 3A**).

**Figure 6:**
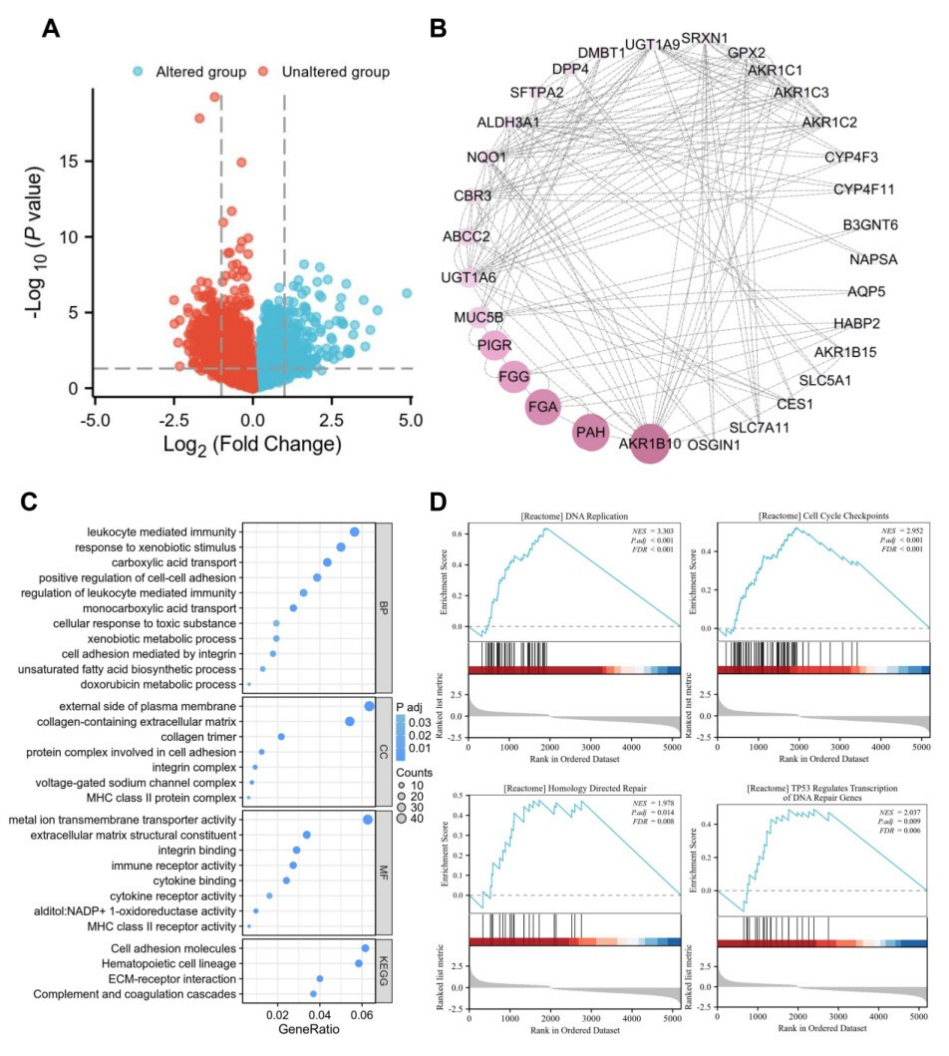
Co-expressed genes and pathway enrichment of PRDX1. (A) Volcano plot showing genes positively and negatively correlated with PRDX1 expression obtained from cBioportal website. (B) PPI network plot created by cytoscape software showing the interaction network of the top 84 genes correlated with PRDX1. (C) Bubble plot showing GO (including BP, CC, MF) and KEGG pathway enrichment of genes significantly correlated with PRDX1 expression. (D) The GSEA pathway, which is positively correlated with PRDX1, is enriched for DNA replication, cell cycle checkpoints, homology directed repair, TP53 regulates transcription of DNA repair genes. NES, normalised enrichment scoring.

To further elucidate the role of PRDX1 in the progression of LUAD, we obtained its co-expressed genes and genetic alteration status based on TCGA-LUAD data by accessing the cBioportal website. The results indicated that 28 out of 503 (6%) patients exhibited PRDX1 alterations. Among all genetic alteration types, mRNA overexpression accounted for a substantial proportion (**Supplementary Figure 3B**). The co-expressed genes identified based on PRDX1 mutations were presented in the volcano plot (**Figure 6A; Supplementary Table 1**). A total of 688 genes were significantly positively or negatively correlated with PRDX1. By setting a threshold of |log_2_FC| > 1.8 and *P* < 0.05, we identified the top 84 genes that were positively or negatively correlated with PRDX1. Their interactions were visualized using protein-protein interaction (PPI) network diagrams (**Figure 6B**). To explore the biological functions of PRDX1 in LUAD, we conducted a functional enrichment analysis of genes that showed significant positive or negative correlations with PRDX1. Biological process (BP) enrichment was associated with leukocyte-mediated immunity, cell adhesion mediated by integrins, and xenobiotic metabolic processes. Enriched cellular component (CC) was associated with the external side of the plasma membrane, collagen-containing extracellular matrix (ECM), and protein complex involved in cell adhesion. Enriched molecular function (MF) was associated with metal-ion transmembrane transporter activity, ECM structural constituents, cytokine receptor activity, and MHC class II receptor activity receptor activity. In addition, KEGG pathway enrichment analysis showed that PRDX1 expression correlated with cell adhesion molecules, ECM-receptor interaction, and complement and coagulation cascades (**Figure 6C**). GSEA revealed a positive correlation between PRDX1 expression and gene sets enriched for DNA replication, cell cycle checkpoints, homology-mediated repair, and DNA repair genes regulated by TP53. These results suggest that PRDX1 is involved with DNA replication, DNA repair, cell adhesion, and cell proliferation processes in LUAD (**Figure 6D**).

### Impact of PRDX1 silencing on the sensitivity of LUAD cells to osimertinib

**Figure 7:**
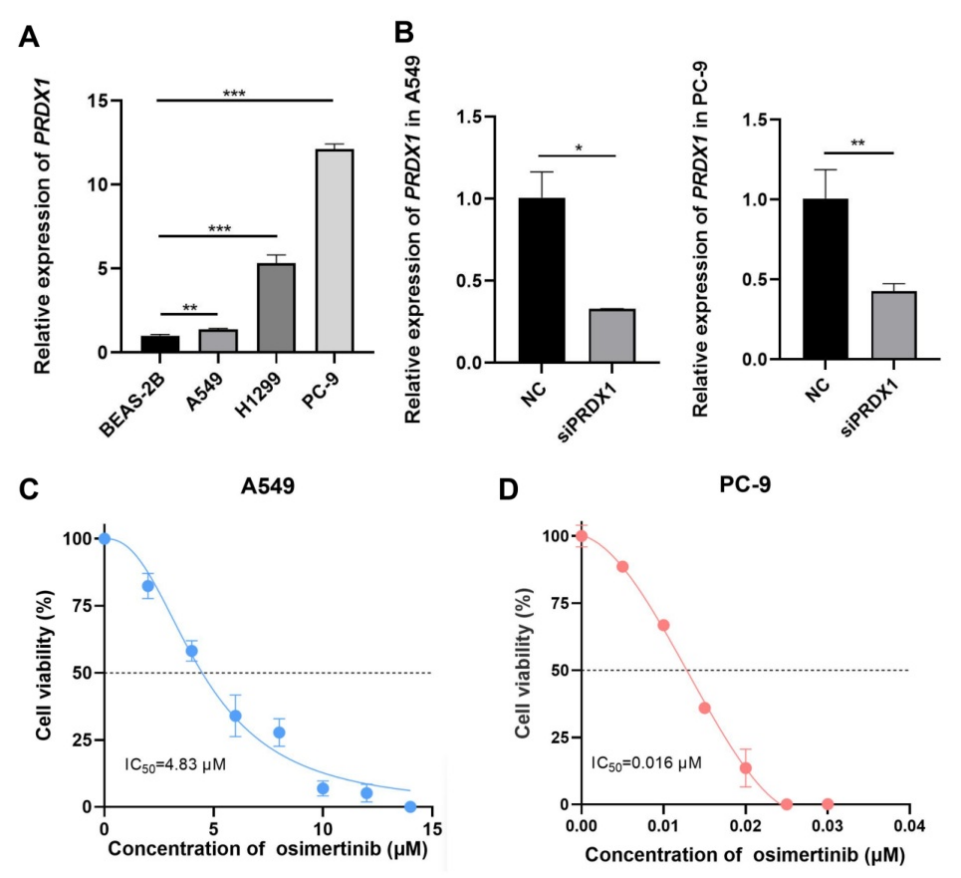
Detection of expression of PRDX1 in LUAD cells and the IC_50_ of LUAD cells for osimertinib. (A) Relative expression of PRDX1 in different LUAD cell lines and in human normal lung epithelial cells, U6 was used as the nuclear internal reference. (B) mRNA knockdown efficiency of PRDX1. (C) Dose-dependent cytotoxicity of osimertinib in A549 cell lines. (D) Dose-dependent cytotoxicity of osimertinib in PC-9 cell lines (OD value=450 nm). Data are presented as mean ± SD; *p < 0.05, **p < 0.01, ***p < 0.001 by Student's *t*-test.

**Figure 8:**
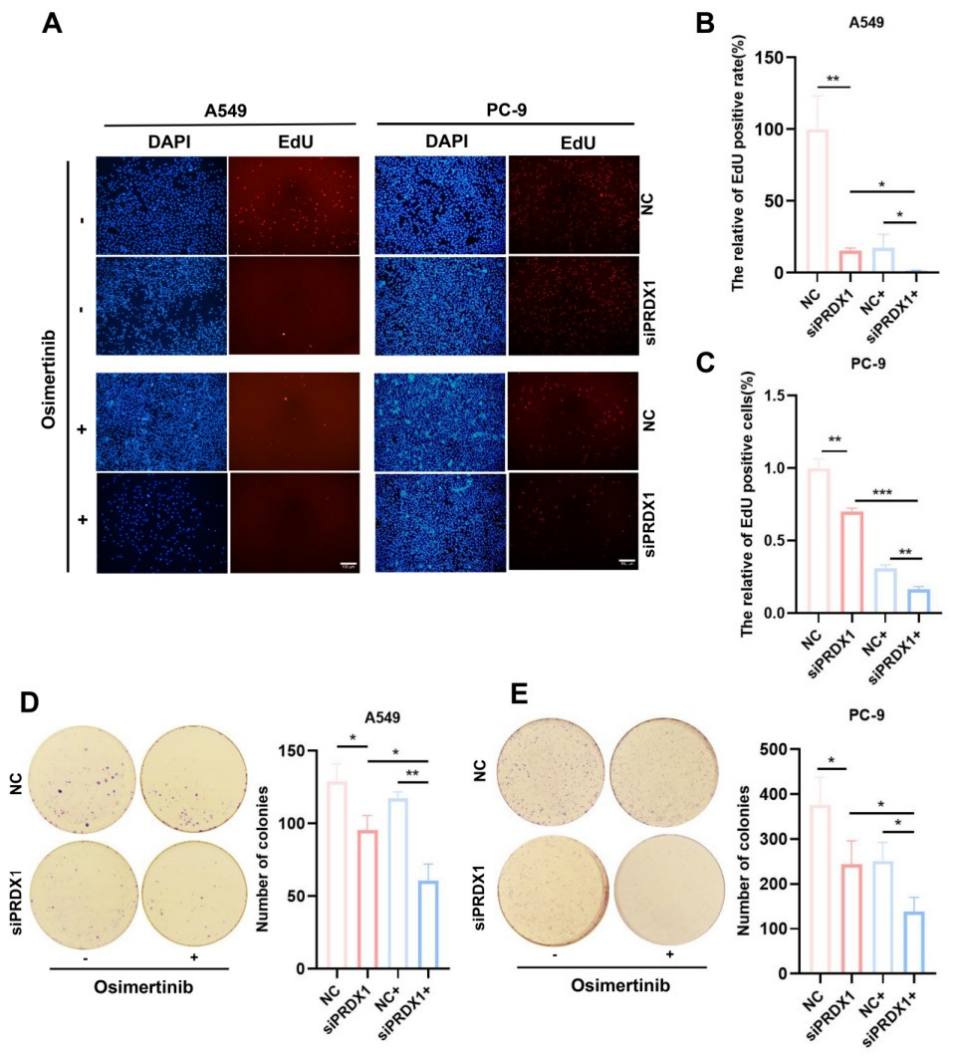
Impact of PRDX1 knockdown on the sensitivity of EGFR-mutant and wild-type LUAD cells for osimertinib. (A-E) Impact of PRDX1 on the sensitivity of A549 and PC-9 cells for osimertinib was assessed by proliferation activity through EdU uptake assays (Scale bar: 100 μm), and colony formation test (colonies >50 μm counted as positive). Data are presented as mean ± SD; *p < 0.05, **p < 0.01, ***p < 0.001 by Student's *t*-test.

To further validate the association between PRDX1 and EGFR mutations, we examined the expression of PRDX1 in human normal lung epithelial cells (BEAS-2B) and LUAD cell lines (A549, PC-9 and H1299). As depicted in **Figure 7A**, the mRNA level of PRDX1 was elevated in A549, PC- 9 and H1299 cells compared to BEAS-2B cells. Notably, PRDX1 expression in EGFR-mutant cells (PC-9) was approximately 12-fold higher than that in wild-type cells (A549). The knockdown efficiency of PRDX1 in the A549 and PC-9 cell lines was verified by qRT-PCR (**Figure 7B**). We further explored the relationship between PRDX1 and the chemosensitivity of LUAD to osimertinib using these two cell lines. In the drug assay, CCK-8 was used to determine the IC_50_ of 4.83 μM (**Figure 7C**) and 0.016 μM (**Figure 7D**) of osimertinib in the A549 and PC-9 cell lines, respectively. To assess the impact of PRDX1 knockdown on the proliferative capacity of LUAD cells, we used siRNA to knock down the expression of PRDX1 in A549 and PC-9 cells. The EdU assay showed that the proliferative activity of A549 and PC-9 cells was diminished after PRDX1 knockdown (**Figure 8A-C**). The colony formation assay showed that the colony formation ability of A549 and PC-9 cells was reduced following PRDX1 downregulation (**Figure 8D-E**). In addition, A549 and PC-9 cells with PRDX1 knockdown were incubated with or without osimertinib, and the effect of PRDX1 knockdown on the efficacy of osimertinib in LUAD was evaluated using EdU and colony formation assays. The results indicated that PRDX1 knockdown significantly enhanced the inhibitory effect of osimertinib on the proliferative and colony formation abilities of LUAD cells. This finding suggests that the knockdown of PRDX1 acts synergistically with osimertinib in inhibiting LUAD proliferation.

**Figure 9:**
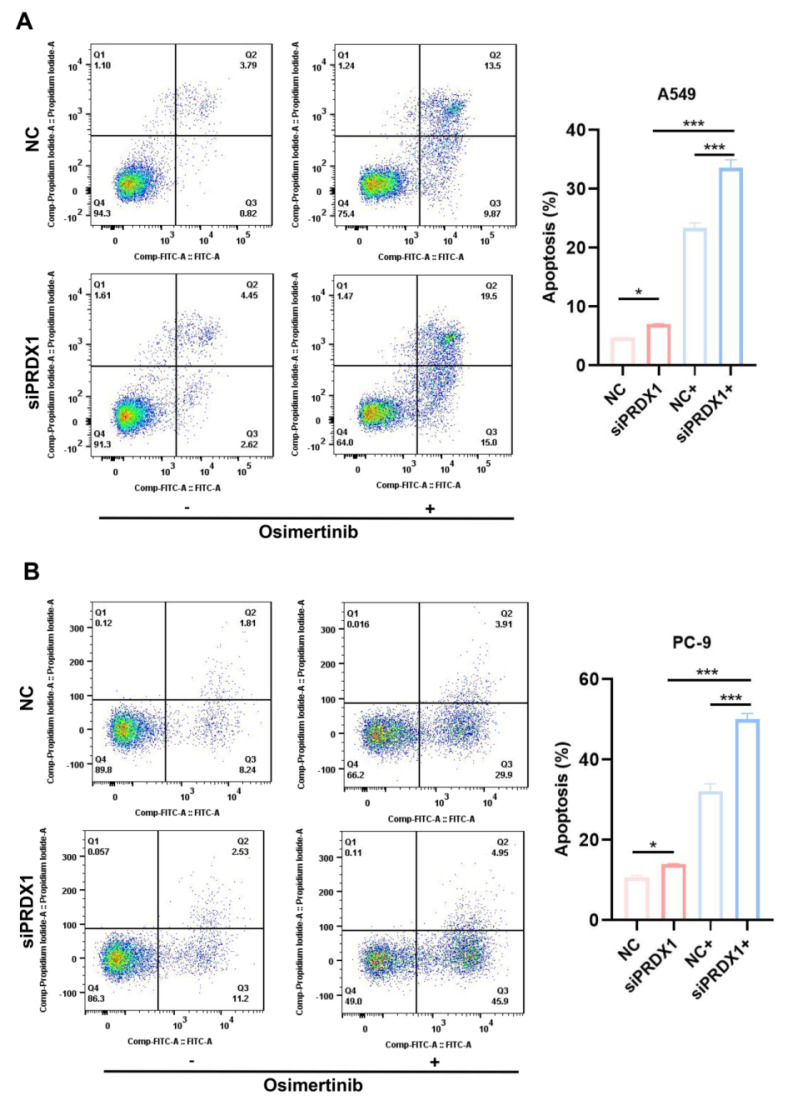
Effect of presence/absence PRDX1 on osimertinib-induced apoptosis of EGFR-mutant and wild-type LUAD cells. (A) Detection of PRDX1 knockdown on osimertinib- induced apoptosis of A549 cells. (B) Detection of PRDX1 knockdown on osimertinib-induced apoptosis of PC-9 cells. Data are presented as mean ± SD; *p < 0.05, **p < 0.01, ***p < 0.001 by Student's *t*-test.

The apoptosis assay showed that the proportion of apoptotic cells among PRDX1 knockdown A549 cells incubated with osimertinib was 10.16% higher than that in A549 cells incubated with osimertinib alone (**Figure 9A**). In contrast, when osimertinib was added, the proportion of PRDX1 knockdown PC-9 cells was 17.88% higher than that in control group (**Figure 9B**). The synergistic pro-apoptotic effect of PRDX1 knockdown and osimertinib was more prominent in EGFR-mutant cells than in wild-type cells, highlighting its therapeutic potential for EGFR-driven LUAD. In conclusion, these results suggest that the combination of PRDX1 knockdown and osimertinib treatment significantly inhibits proliferation and promotes apoptosis of LUAD cells. Compared to A549 cells, silencing PRDX1 promotes the sensitivity of PC-9 cells to osimertinib, further demonstrating that inhibition of PRDX1 synergizes the ability of osimertinib to suppress the progression of EGFR-mutant LUAD.

### Association between the expression of PRDX1 and immune cell infiltration in LUAD TME

**Figure 10:**
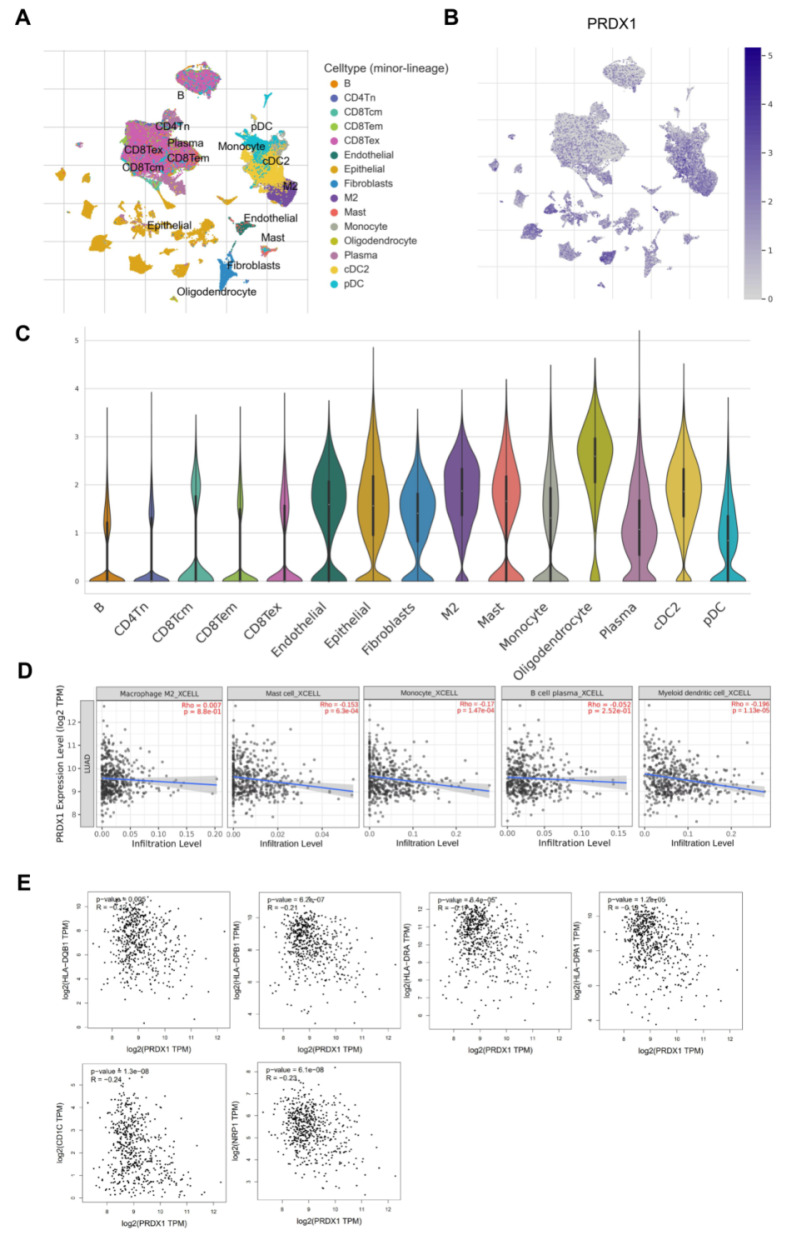
Association between the expression of PRDX1 and immune cells infiltration**.** (A) TME cell types and corresponding cell distributions in the GSE131907 set were obtained from the TISH database. (B**)** Distribution of PRDX1 in various cell types of the TME at single-cell resolution. (C) Violin plots demonstrating PRDX1 expression in various cell types of TME. (D) Correlation of PRDX1 expression with the infiltration of M2 macrophages, mast cells, monocytes, plasma cells, and DCs obtained using the TMIER2.0 website tool. (E) Correlation of PRDX1 expression with molecular markers of DCs, including HLA-DQB1, HLA-DPB1, HLA-DRA, HLA-DPA1, CD1C, and NRP1. Correlation analyses were statistically performed using the Spearman algorithm.

The TME plays an important role in tumor development 34. To further explore the correlation between PRDX1 and the TME in LUAD, we retrieved the GSE131907 dataset from the TISCH database and carried out a comprehensive investigation. **Figure 10A** illustrates the type and distribution of immune cells within this dataset. Meanwhile, **Figure 10B** presents the distribution of PRDX1 across different clusters of TME-associated cells at the single-cell resolution. **Figure 10C** depicts the expression of PRDX1 in each TME cell type using violin plots. PRDX1 expression was elevated in epithelial cells, mesenchymal cells, oligodendrocytes, plasma cells, and innate immune cells. The latter category includes DCs (both plasmacytoid dendritic cells [pDC] and conventional dendritic cells 2 [cDC2]), mast cells, monocytes, and M2 macrophages. Among these cell types, oligodendrocytes exhibited the highest PRDX1 expression, followed by cDC2. These results support the notion that PRDX1 is closely associated with the tumor immune microenvironment (TIM) of LUAD.

Subsequently, we utilized the TIMER2.0 website to analyze the correlation between PRDX1 expression and the infiltration of immune cells with relatively high PRDX1 expression, as previously described. The results indicated that PRDX1 had a significant negative correlation with mast cell, monocyte, and DC infiltration (**Figure 10D**). Considering that PRDX1 showed the strongest negative correlation with DC infiltration, we further investigated the correlation between PRDX1 expression and various immune markers in DCs. These markers included HLA-DQB1, HLA-DPB1, HLA-DRA, HLA-DPA1, CD1C, and NRP1. As shown in** Figure 10E**, PRDX1 was negatively correlated with each immune marker in DCs. In conclusion, these results suggest that PRDX1 is closely associated with the infiltration of immune cells in LUAD, particularly the degree of DC infiltration. DCs play a role in adjuvant anti-tumor immunity within the TME. Therefore, targeting PRDX1 may present a novel strategy for enhancing the efficacy of immunotherapy.

## Discussion

LUAD, a major subtype of NSCLC, currently faces significant limitations in its early diagnosis and treatment strategies 35, 36. Thus, there is a pressing need to explore more biomarkers with diagnostic and prognostic value. The PRDX family genes are expressed in various tissues of the human body. The family is involved in a wide spectrum of tumorigenesis and tumor progression processes, holding the potential to serve as molecular markers and therapeutic targets. For example, Ying et al. 37 found that PRDX1 could enhance tumor invasion and metastasis through its interaction with LINC00460. However, the functions of PRDX family members in LUAD remain unclear and require systematic investigation.

We employed bioinformatic tools to systematically evaluate the prognostic value of each member of the PRDX family, as well as their differential expression between LUAD samples and normal tissues. At both the transcriptional and protein levels, PRDX1, PRDX2, PRDX3, and PRDX6 were highly expressed in LUAD tissues. Furthermore, except for PRDX5, all PRDX genes exhibited a trend of significantly elevated expression as tumor progression and lymphatic metastasis occurred. Additionally, high expression of PRDX1, PRDX2 and PRDX3 was associated with poor OS and FPS. These results indicate that high expression of PRDX1, PRDX2, PRDX3, and PRDX6 is closely associated with the progression of LUAD. Monitoring the expression status of these genes can predict the clinical features of LUAD. Among them, PRDX1, PRDX2 and PRDX3 can potentially serve as prognostic biomarkers for LUAD, and further exploration of their biological functions is warranted.

Drug sensitivity data for each PRDX family member indicated that the mRNA expression of PRDX1, PRDX4, and PRDX6 was positively correlated with the IC_50_ of the top 30 representative drugs in the CTRP database. This finding suggests that high expression of these genes may mediate drug resistance, particularly to BIX-01294, a histone methyltransferase inhibitor known to exert anti-tumor effects in various tumors 38. Ji et al. 32 reported that BIX-01294 induced apoptosis by inhibiting BCKDHA-mediated alteration of mitochondrial metabolism in EGFR-mutated LUAD. These results inspired us to explore the expression of PRDX family members with higher prognostic value (PRDX1, PRDX2, and PRDX3) in patients with EGFR-mutated LUAD. Notably, in the GSE31210 dataset, the expression of PRDX1 was significantly higher in EGFR-mutant LUAD compared to the wild-type, while no such difference was observed for PRDX2 and PRDX3. This finding reveals the oncogeneic role and biomarker potential of PRDX1 in patients with EGFR-mutant LUAD. By integrating the results of expression analysis of the PRDX family, evaluation of prognosis, studies on methylation, assessment of drug sensitivity, and the association with EGFR mutations, we decided to further investigate the role of PRDX1 in the progression of LUAD.

Based on the established association between PRDX1 and EGFR mutations, we delved deeper into its association with the sensitivity to EGFR-TKI drugs. Osimertinib, a third-generation EGFR-TKI, has been demonstrated to effectively extend the OS of patients with advanced LUAD 33. Nevertheless, issues such as recurrence and drug tolerance remain unavoidable 39-41. Thus, there is an urgent necessity to explore new targets to enhance the sensitivity of EGFR-TKI therapy in patients with LUAD. Molecular docking analysis results indicated that the binding energies between PRDX1 and osimertinib were -5.83 and hydrogen bonding played a role in the formation of the complex. This suggests a potential direct interaction between the two. Analysis of clinical samples revealed that PRDX1 expression was higher in 13 EGFR-mutant (exon 21 mutation) samples compared to that in 12 EGFR wild-type samples. Therefore, we postulated that PRDX1 might serve as a potential target for enhancing the sensitivity of EGFR-TKI.

To further elucidate the tumor-promoting mechanism of PRDX1, we used functional enrichment analysis to uncover its multi-pathway regulatory network. GO and KEGG analysis indicated that PRDX1 expression was closely associated with cell adhesion mediated by integrin, cytokine receptor activity, and ECM-receptor interaction. These pathways play a vital role in facilitating tumor growth and metastasis 42-45. This suggests that PRDX1 is implicated in the crucial process underlying the development of LUAD. Moreover, GSEA revealed that PRDX1 expression was positively correlated with DNA replication, cell cycle checkpoints, homology-mediated repair, and DNA repair genes regulated by TP53. As DNA repair is intricately connected to the development of drug resistance in tumor cells 46, it could be inferred that PRDX1 may influence the sensitivity of EGFR-TKI treatment in LUAD patients via genomic instability.

Therefore, we conducted *in vitro* experiments to verify the association between PRDX1 expression and the sensitivity of EGFR-TKI treatment. Owing to the presence of EGFR mutations, PC-9 cells exhibited higher sensitivity to EGFR-TKI. The IC_50_ of osimertinib for PC-9 cells was 0.016 μM. Conversely, the A549 cell line was EGFR wild-type, and its osimertinib IC_50_ was 4.83 μM. Furthermore, functional experiments demonstrated that PRDX1, acting as an oncogene, plays a crucial role in the growth and proliferation of LUAD. Inhibiting its expression can suppress the proliferation of LUAD cells and promote cell apoptosis. Notably, knocking down PRDX1 led to a 17.88% increase in the apoptosis rate of EGFR-mutant cells incubated with osimertinib, while the apoptosis rate of wild-type cells incubated with osimertinib increased by only 10.16%. This finding further supports the conclusion that the sensitivity of EGFR-mutant cells to osimertinib markedly increased after PRDX1 inhibition compared to EGFR wild-type cells, suggesting that inhibition of PRDX1 has a synergistic effect with osimertinib in suppressing tumor growth.

In addition, a connection exists between EGFR mutations and the TME. For instance, in 2021, Chen et al. 47 discovered that EGFR could trigger T-cell immune dysfunction by activating immunoglobulin-like transcript 4 (ILT4), thereby mediating the immune escape of NSCLC cells. Moreover, PRDX1 has been reported to regulate the balance of Th1/Th2 phenotypic transition in CD4+ T cells 48. Single-cell analysis revealed that PRDX1 expression was the highest in oligodendrocytes, followed by cDC2. Notably, high PRDX1 expression was negatively correlated not only with DC infiltration but also with the molecular characteristics of DCs. Considering the pivotal role of DCs in activating anti-tumor T cells 49, further investigations into the association between PRDX1 and DC subtypes may offer novel strategies for DC-based anti-tumor therapy.

## Conclusion

Through systematic bioinformatic investigations into the expression of PRDX family members, we identified that elevated PRDX1 expression is associated with poor prognosis in patients with LUAD. Furthermore, PRDX1 expression is not only correlated with EGFR mutation status but also with the sensitivity of EGFR-TKI treatment. The knockdown of PRDX1 exerts a synergistic effect when combined with EGFR-TKI treatment, potently suppressing tumor growth. Additionally, there is a significant association between PRDX1 expression and DC infiltration. Further in-depth exploration of the interaction between PRDX1 and DC subtypes could provide novel strategies for enhancing antitumor immunity. Collectively, our findings demonstrate that PRDX1 serves as a potential prognostic biomarker for LUAD. Moreover, PRDX1 may play an important role in modulating EGFR-TKI therapeutic sensitivity and tumor immunity, highlighting its promise as a therapeutic target to improve clinical outcomes in LUAD.

## Supplementary Material

Supplementary figures.

Supplementary table.

## Figures and Tables

**Table 1:** Primer sequence for qRT-PCR

Gene	Primer (Forward)	Primer (Reverse)
PRDX1	CCACGGAGATCATTGCTTTCA	AGGTGTATTGACCCATGCTAGAT
U6	CTCGCTTCGGCAGCACA	AACGCTTCACGAATTTGCGT

## Abbreviations

LUAD: lung adenocarcinoma
PRDX: peroxiredoxin
EGFR-TKI: epidermal growth factor receptor-tyrosine kinase inhibitor
EGFR: epidermal growth factor receptor
TKI: tyrosine kinase inhibitor
IC₅₀: half-maximal inhibitory concentration
CCK-8: Cell Counting Kit-8
EdU: 5-Ethynyl-2'-deoxyuridine
TME: tumor microenvironment
DCs: dendritic cells
NSCLC: non-small cell lung carcinoma
SCC: squamous cell carcinoma
OSCC: oral squamous cell carcinoma
TCGA: The Cancer Genome Atlas
TPM: transcripts per million
IHC: immunohistochemistry
HPA: Human Protein Atlas
RNA-seq: RNA sequencing
OS: overall survival
FPS: first progression survival
CTRP: The Cancer Therapeutics Response Portal
GSCA: Gene Set Cancer Analysis
GO: Gene Ontology
KEGG: Kyoto Encyclopedia of Genes and Genomes: GSEA: Gene Set Enrichment Analysis
NES: normalized enrichment score
FFPE: formalin-fixed paraffin-embedded
qRT-PCR: quantitative real-time polymerase chain reaction
FBS: fetal bovine serum
siRNA: small interfering RNA
OD: optical density
PBS: phosphate-buffered saline
PPI: protein-protein interaction
BP: biological process
CC: cellular component
ECM: extracellular matrix
MF: molecular function
pDC: plasmacytoid dendritic cells
cDC2: conventional dendritic cells 2
TIM: tumor immune microenvironment
ILT4: immunoglobulin-like transcript 4
