# Supplementary material for: Malignant features related PRDX1 associated with osimertinib sensitivity of EGFR-mutant lung adenocarcinoma
Source: Int J Med Sci. 2025 Mar 31;22(9):2040–58. doi: 10.7150/ijms.107255 (PMC12035832; doi:10.7150/ijms.107255)
Supplement: Supplementary file 1 — Supplementary figures. [file ijmsv22p2040s1.pdf]

Supplementary Figures

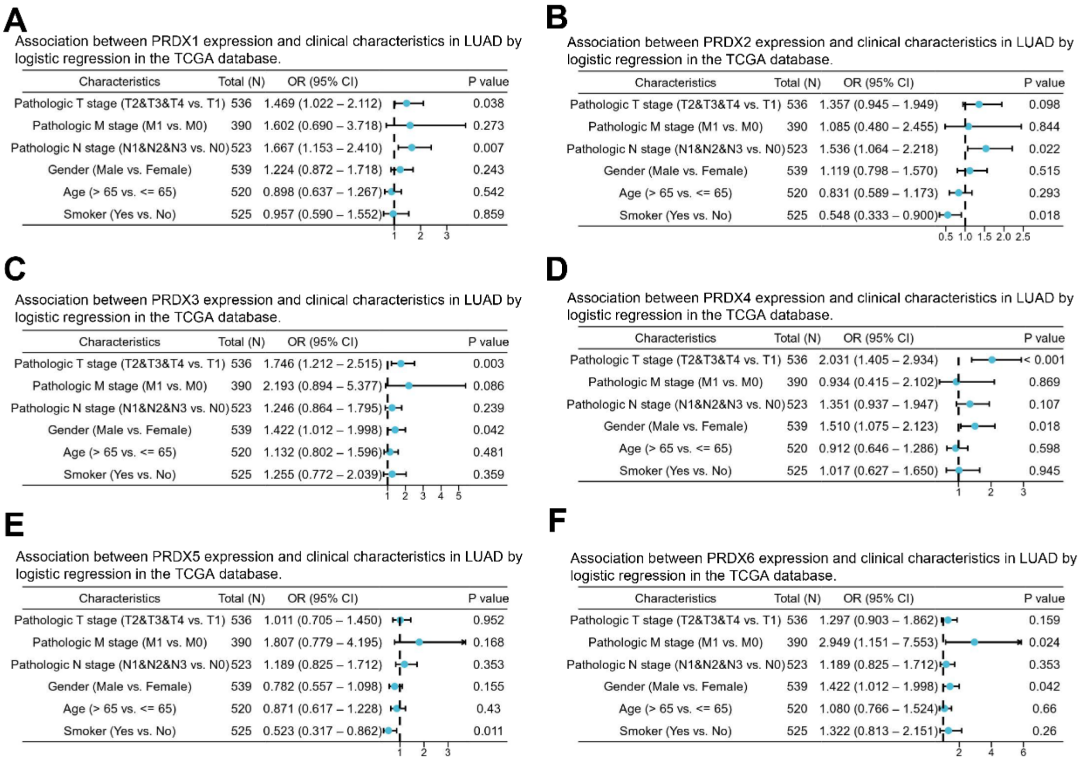

Supplementary Figure 1: Association between PRDX1-6 expression and clinical characteristics in LUAD. (A-F) Correlation between PRDX1-6 expression and clinical characteristics in LUAD analyzed by logistic regression in the TCGA database.

A

Characteristics	Total(N)	HR(95% CI) Univariate analysis	P value Univariate analysis
Pathologic T stage	527		
T1	176	Reference	
T2	285	1.507 (1.059 - 2.146)	0.023
T3&T4	66	3.095 (1.967 - 4.868)	< 0.001
Pathologic N stage	514		
N0	345	Reference	
N1	96	2.293 (1.632 - 3.221)	< 0.001
N2&N3	73	2.993 (2.057 - 4.354)	< 0.001
Pathologic M stage	381		
M0	356	Reference	
M1	25	2.176 (1.272 - 3.722)	0.005
Gender	530		
Female	283	Reference	
Male	247	1.087 (0.816 - 1.448)	0.569
Age	520		
<= 65	257	Reference	
> 65	263	1.216 (0.910 - 1.625)	0.186
Smoker	516		
No	74	Reference	
Yes	442	0.942 (0.625 - 1.420)	0.775
PRDX1	530		
Low	266	Reference	
High	264	1.352 (1.009 - 1.812)	0.043
PRDX2	530		
Low	265	Reference	
High	265	1.170 (0.878 - 1.559)	0.284
PRDX3	530		
Low	267	Reference	
High	263	1.463 (1.093 - 1.957)	0.01
PRDX4	530		
Low	264	Reference	
High	266	1.218 (0.912 - 1.625)	0.181
PRDX5	530		
Low	262	Reference	
High	268	1.093 (0.820 - 1.457)	0.543
PRDX6	530		
Low	265	Reference	
High	265	1.604 (1.198 - 2.147)	0.001

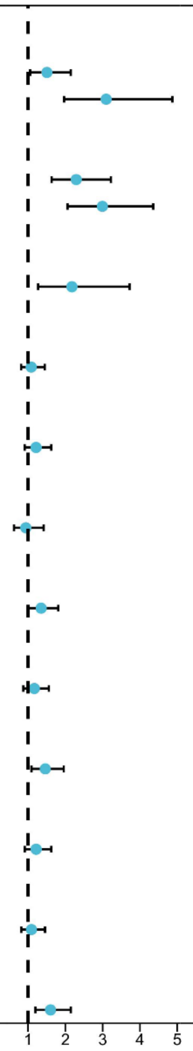

Supplementary Figure 2: Independent prognostic risk factors for OS in patients with LUAD. (A) Independent prognostic risk factors for OS in patients with LUAD identified by univariate regression analysis of TCGA-LUAD dataset.

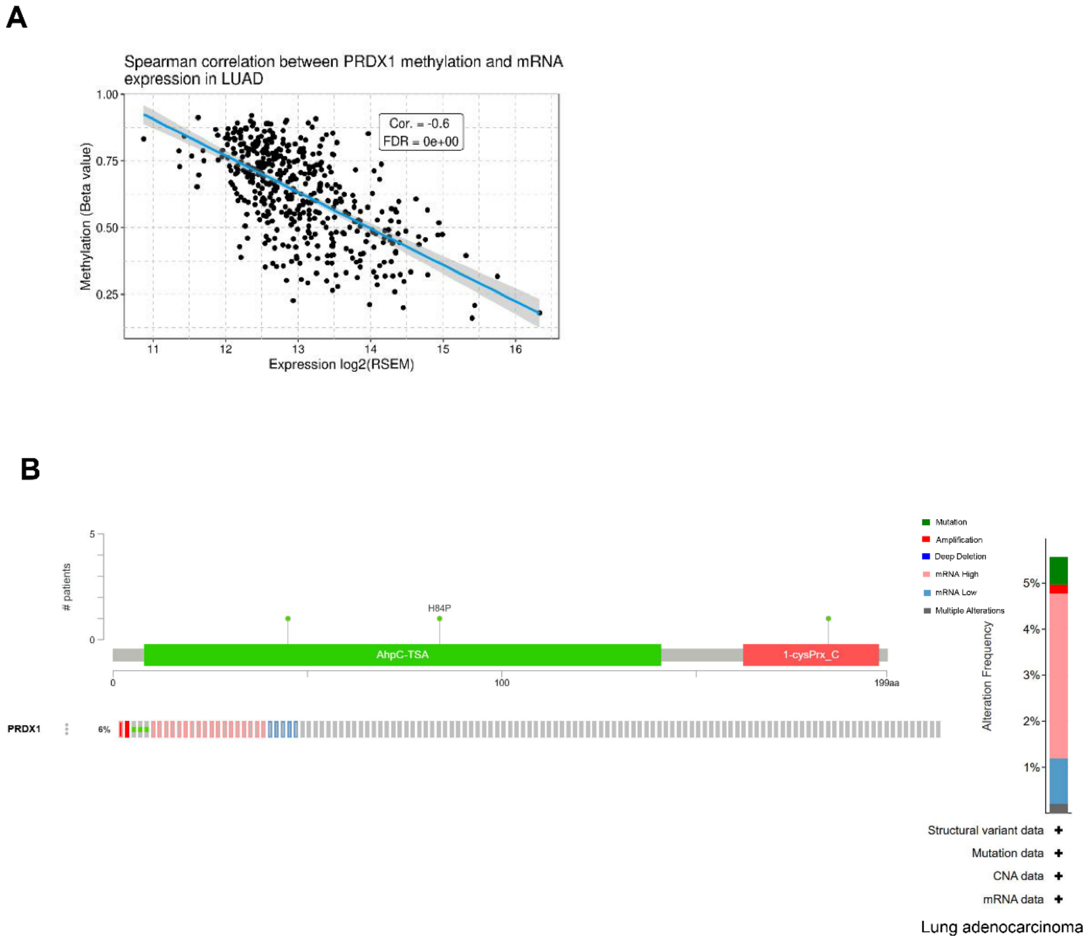

Supplementary Figure 3: Methylation analysis and mutant landscape of PRDX1. (A) Correlation between PRDX1 expression and methylation analyzed in GSCA database. (B) Main mutation sites of PRDX1 genome alterations and mutant landscape of PRDX1 identified in cBioportal website.
